# Epidemiology of potential source, risk attribution of *Clostridium perfringens* from Egyptian broiler farms and genetic diversity of multidrug resistance strains

**DOI:** 10.1038/s41598-025-12519-0

**Published:** 2025-08-05

**Authors:** Rasha Elkenany, Mona Elsayed, Amira Zakaria, Reham Elnagar, Mona Salem, Aya Auob, Amal Awad

**Affiliations:** 1 Department of Bacteriology, Immunology, and Mycology, Faculty of Veterinary Medicine, Mansoura University, Mansoura, 35516 Egypt; 2 Department of Hygiene and Zoonoses, Faculty of Veterinary Medicine, Mansoura University, Mansoura, 35516 Egypt; 3 Department of Food Hygiene and Control, Faculty of Veterinary Medicine, Mansoura University, Mansoura, 35516 Egypt

**Keywords:** *Clostridium perfringens*, Antimicrobial resistance, Toxinotypes, Risk factors, ERIC-PCR, Necrotic enteritis, Microbiology, Molecular biology, Diseases, Risk factors

## Abstract

*Clostridium perfringens* induced necrotic enteritis (NE) became a persistent problem that had a major financial impact on the poultry business worldwide. Nevertheless, no prior research has assessed the related risk factors in Egyptian broiler farms. Thus, the purpose of this study is to perform an epidemiological analysis of *C. perfringens* occurrence, toxinotyping, and risk factors in El-Dakhlia and Damietta provinces, Egypt as well as their characteristics of antimicrobial resistance and molecular typing. From 25 broiler farms, 1100 samples were gathered. Antimicrobial resistance profiles and molecular typing were used to characterize the isolates. The findings revealed an overall prevalence of 26.3% (289/1100) from chickens’ clinical samples (30.3%; 273/900) and farm environmental samples (8%; 16/200). Toxinotyping of 289 isolates showed that 165 (57.1%) isolates were *C. perfringens* type A, harboring only alpha toxin gene (*cpa*) while 124 (42.9%) isolates were *C. perfringens* type G, containing *net*B gene. The *cpb2* genes were found in 66 (22.8%) isolates with the highest positive rate from dead birds. Additionally, the study found a number of possible risk variables that were substantially linked to the prevalence of *C. perfringens*, including location in Damietta, winter season, history of coccidia infection, use of antimicrobial growth promoters, birds older than 22 days, wet litter type, and biosecurity strategy. Univariate and multivariate analyses showed a significant association between *C. perfringens* infection and grower chickens (OR 2.09, 95% CI 1.05–4.21, p = 0.037) compared to starter chickens. The isolates displayed their highest resistance rate to sulfamethoxazole-trimethoprim (94.5%), erythromycin, imipenem (94.1% each), penicillin, ampicillin, streptomycin, gentamycin (90.7% each), ampicillin/sulbactam (89.9%), cefuroxime and cefepime (85.8%), nalidixic acid (85.1%), and tetracycline (78.9%). Remarkably, none of the strains were resistant to meropenem. Multidrug-resistant was observed in 94.5% strains with MAR index of 0.32–0.79. The resistance genes carried by different strains were obviously different, among which the detection rate of aminoglycoside resistance gene *aphA1*, was the highest (100%), followed by *bla*_TEM_ (85.8%), *erm*B, *qnr*S (85.1%), *sul*1 (80.6%), *tet*A(78.9%), *drf*A-1 (75.1%), *qnr*D, *sul*2 (60.2%), *cat*A (57.4%), *aad*A (57.4%), *amp*C, *mef*A, *qnr*A (50.2%), *bla*_CTX_ (46%), and *tet*M (42.2%). Enterobacterial repetitive intergenic consensus polymerase chain reaction (ERIC-PCR) was used to classify these isolates into eight different genotypes according to sampling place and sample type. The epidemiological information from this study was helpful in determining the danger of clostridial infection linked to Egyptian broiler farms. Our results also show that in order to combat multidrug resistance, new medications and antibiotic substitutes are required. The importance of conducting more surveys to better understand the prevalence of *C. perfringens* infection under strict management circumstances for various flock purposes cannot be overstated.

## Introduction

*Clostridium perfringens* is an anaerobic, spore-forming, Gram-positive bacterium that causes necrotic enteritis (NE) in broiler chickens^1^. On poultry farms, *C. perfringens* can be isolated from soil, bird droppings, litter, live insects, poultry feed, water pipes, nipple-drinker drip cups, floors, walls, fans, and farm workers’ clothing and shoes^2^. *C. perfringens*spores pose a serious danger of infection and reinfectiondue to their extreme resistance to desiccation, chemicals, and temperature^1^. Hence, pathogenic strains of *C. perfringens* could spread vertically from hens to newly hatched chicks, through the fecal–oral route, and through contaminated food, water, housing structures, and insects^2^. The expansion of pathogenic *C. perfringens*, which results in intestinal mucosal necrosis, causes necrotic enteritis in chicken. The subclinical form of NE is more significant, even when clinical outbreaks may result in high fatality rates. Because the disease goes unnoticed, sick birds go untreated, causing significant financial losses for the poultry sector^3^. Acute clinical or moderate subclinical enterotoxemia are the two possible outcomes of NE outbreaks, which are the culmination of a complex chain of events^4^. Nevertheless, the occurrence of a pathogenic strain of *C. perfringens* alone is insufficient to cause this illness since predisposing variables are required to create the ideal environment for its growth^5^. The development of NE induced by *C. perfringens* may be influenced by a number of predisposing factors. The digesta’s thickening, seasonal fluctuations, the dietary composition (fishmeal or wheat and barley, antimicrobial and anticoccidial, animal protein and soy content), the age of the birds, the presence of coccidiosis, wet litter, the use of ammonia as a disinfectant, plasterboard walls, overcrowding, and stress are some of these factors^4,6,7^.

The generation of many poisons and extracellular enzymes is necessary for *C. perfringens* to be virulent^8^. Despite this, the species *C. perfringens* produces over 15 distinct toxins that cause a range of animal diseases. According to the presence of genes encoding the toxins CPA, CPB, ETX, and ITX, as well as the newly added enterotoxin CPE and pore-forming necrotic enteritis B-like toxin (netB), strains of *C. perfringens* are categorized into seven distinct toxinotypes, ranging from A to G^9^. *C. perfringens* toxinotype A strains are primarily responsible for NE in poultry, producing phospholipase C (PLC or α toxin) and netB among other toxins^1^. Type C also contributes to NE to a lesser degree. According to Uzal et al.^10^, the novel type *C. perfringens* toxinotype G is the cause of NE in chicken and produces the CPA and netB toxins. The *cpa* gene, which codes for PLC, is found on the chromosome of every strain of *C. perfringens* and exhibits only slight sequence differences^1^. Due to PLC’s hydrolysis of cell membrane lecithin, RBCs, platelets, and mucosa are destroyed, resulting in intestinal mucosal necrosis. Conversely, it has been demonstrated that a pathogenic strain of *C. perfringens* with an inactivated *cpa* gene may nevertheless cause NE lesions that are similar to those observed in birds challenged with the wild-type strain, indicating that PLC is not the primary virulent factor in this illness^11^. Avian virulent *Cgene,fringens* strains associated with NE secrete a pore-forming toxin, referred to as netB toxin; encoded by *net*B gene; which is essential for causing NE in chickens^11^. A large clostridial glucosylating cytotoxin referred to as *C. perfringens* TpeL, also appears to have a role in the pathogenesis of NE^12^ and potentiates the effect of other *C. perfringens* virulence factors associated with NE. Other Toxins as chromosomally encoded bacteriocin (perfrin), glycohydrolases, metalloprotease, leukocidins, internalin, and putative adhesins can be involved in some NE-inducing strains^13^.

Due to the extensive use of antibiotics in poultry in recent years, antimicrobial resistance strains of *C. perfringens* have been appearing daily in different regions^14^. This has led to a gradual rise in the incidence of clostridial infection in poultry as well as a decrease in the effectiveness of antimicrobial agents. In addition to creating significant challenges for veterinary therapeutic care, it poses a major risk to food safety^1^. *C. perfringens* was found to be resistant to penicillin and its substitutes, including imipenem, metronidazole, ceftriaxone, and clindamycin, in a prior study^15^. Additionally, strains of *C. perfringens* have been found to be resistant to tetracycline, lincomycin, enrofloxacin, cefoxitin/ampicillin, and erythromycin by the identification of the *tet*, *Inu*, *qnr*, *bla*, and *erm*(B) genes, respectively^16^. Most acquired antimicrobial resistance genes are linked to plasmids. However, many studies focus solely on the phenotypic signs of antibiotic resistance^17^. Therefore, more research is required to ascertain *C. perfringens’s* resistance genetic profile status.

The enterobacterial repetitive intergenic consensus sequence-based PCR (ERIC-PCR) has been used to investigate the genetic diversity of *C. perfringens* isolates and its traceability from the perspective of epidemiological surveillance and bacterial genotypic diversity^18^. For comparing and examining the DNA fingerprinting patterns of *C. perfringens* strains, the ERIC-PCR is quicker and less expensive. ERIC is now regarded as the main technique in disease surveillance, disease clustering, and outbreak investigation programs. A useful and sensitive method for determining the genetic differentiation of *C. perfringens* isolated from various sites is the ERIC sequences, which are largely conserved at the nucleotide sequence level but differ in chromosomal positions and numbers among species.

No prior research has assessed the associated risk factors and scarce information is available on the drug resistance mechanisms and molecular typing of *C. perfringens* isolated from Egyptian broiler farms in Dakahlia and Damietta Governorates, Egypt. Therefore, the primary goals of this research were (1) to systematically survey the prevalence of *C. perfringens* and its associated risk factors in Egyptian broiler farms (2) to molecularly characterize *C. perfringens* toxin genes using multiplex PCR to identify the *cpa, cpb, cpb2, etx, itx, cpe,* and *tpL* genes, as well simplex PCR assays to identify the *net*B gene, (3) to clarify the phenotypic and genotypic antibiotic resistance characteristics of *C. perfringens* isolates, (4) to explore the molecular types using Enterobacterial repetitive intergenic consensus polymerase chain reaction (ERIC-PCR).

## Results

### Prevalence of *Clostridium perfringens*

The prevalence of *C. perfringens* was examined in the current investigation in samples taken from chickens’ clinical samples (cloacal swabs, intestinal scrapings, liver, and feces) and environmental samples (litter, water, feed, feeder, drinker, wall, fans, and workers hand swabs) from broiler farms at El-Dakhlia and Damietta provinces in Egypt. Of the 1100 samples, 289 (26.3, 95% CI 23.69–28.90%) yielded *C. perfringens* depending on microbiological characters and presence of alpha toxin gene (*cpa*), which is characteristic to all *C. perfringens* strains/types. *C. perfringens* strains were more prevalent in chickens’ clinical samples (30.3%, 273/900, 95% CI 27.33–33.34%) involving; 41% (82/200) of intestine, 41% (82/200) liver, 25.6% (64/250) of cloacal swabs, 18% (45/250) of feces; than environmental samples (8%, 16/200, 95% CI: 4.24–11.76%) (11-samples from the litter, 3-samples from the feed, 1-sample from the wall swabs, 1-sample from the workers hand). Of chickens’ clinical samples, 45/250 (18%, 95% CI 13.24–22.76%) isolates were obtained from apparently healthy chicken, 64/250 (25.6, 95% CI 20.19–31.01%) from suspected live chicken, and 164/400 (41, 95% CI 36.18–45.82%) from dead chickens (Table 1). Collectively, statistical analysis using Chi-square test revealed a significant difference (p < 0.001) between the occurrence of *C. perfringens* in chickens’ clinical samples and environmental samples (Table 1).

**Table 1:** Prevalence and distribution of Clostridium *perfringens* (n = 289) in the broiler farms (n = 25).

Sample source		Sample type	No. of samples	No. positive samples (%)	95% CI	P-value	Toxin gene(n)
Chickens’ clinical samples	Suspected live chicken	Cloacal swab	250(10each-farm)	64(25.6%)	20.19–31.01	< 0.001	*cpb*2 (6),*net*B(12)
	Apparently healthy chicken	Feces	250(10each-farm)	45(18%)	13.24–22.76		netB(4)
	Dead chicken	Intestine	200	82(41%)	36.18–45.82		*cpb*2 (30),*net*B(54)
		liver	200	82(41%)			*cpb*2 (30),*net*B(54)
	Total		900	273(30.3%)	27.33–33.34		*cpb*2 (66),*net*B(124)
Environmental samples		litter	25	11(16%)	4.24–11.76		–
		water	25	0			–
		feed	25	3(12%)			–
		Feeder swab	25	0			–
		Drinker swab	25	0			–
		Wall swab	25	1 (4%)			–
		Fan swab	25	0			–
		Workers hand swab	25	1(4%)			–
		Total	200	16(8%)			–
Overall total			1100	289(26.3%)	23.69- 28.90		*cpb*2 (66),*net*B(124)

*CI* confidence interval.

### Toxin-typing of *C. perfringens* isolates

All *cpa*-positive isolates (n = 289) were toxinotyped and tested for the existence of *cpb, cpb2, etx, iap, cpe, tpe*L, and *net*B-toxin genes by the PCR tests. Of the 289 isolates, 66 (22.8%) isolates were positive for the gene encoding the beta2 toxin (*cpb2*), and 124 (42.9%) isolates harbored the *net*B gene, which was classified as type G (Table 1). Of the sixty-six *cpb2*-positive isolates from Chickens’ clinical samples, 60 isolates were recovered from dead chicken, and the remaining six isolates were found in suspected live chicken. These *net*B-positive isolates were obtained from Chickens’ clinical samples (108 from dead chicken, 12 suspected live chicken, and 4 apparent healthy chicken). The *cpb2* gene was found in *net*B-positive isolates. No other *C. perfringens*- associated toxin genes (*cpb, etx, iap, cpe, tpe*L) were found in the isolates from Chickens’ clinical or environmental samples. After testing negative for other toxinotype-defining genes, all identified *Clostridium perfringens* isolates were classified as toxinotype A (57.1%; n = 165/289), originating from both chickens’ clinical samples (n = 149) and environmental samples (n = 16). Among the clinical samples, only two *C. perfringens* toxinotypes—A and G—were detected within the same farm flocks (n = 22). In contrast, isolates from the remaining three farm flocks revealed only toxinotype A.

### Risk factors associated with *C. perfringens* infection

Univariate analysis was conducted to determine the associations between the farm-level prevalence of *C. perfringens* infection and various factors related to the birds, farm management, biosecurity measures, and general farm information (Table 2). In this analysis, twelve variables were significantly correlated with the farm-level *C. perfringens* positive status, whereas the rest of the factors (housing system, farm size, and farm breed) were found to be non-significant with the farm-level outcome status. In the univariable analysis, significantly associated variables for *C. perfringens* infection related to bird, farm management, and biosecurity practices were location in Damietta (OR 1.56; 95% CI 1.19–2.04; p = 0.001), seasonal prevalence in winter (OR 4.32; 95% CI 2.82–6.62; p < 0.001), history of coccidia infection (OR 3.81; 95% CI 2.70–5.38; p < 0.001), use of antimicrobial growth promoters (OR 3.67; 95% CI 2.74–4.91; p < 0.001), birds older than 22 days (OR 3.05; 95% CI 2.26–4.12; p < 0.001), wet litter type (OR 3.84; 95% CI 2.83–5.20; p < 0.001), disinfection of drinking water (OR 0.25; 95% CI 0.19–0.33; p < 0.001), feeder and drinker wash daily (OR 0.32; 95% CI 0.24–0.43; p < 0.001), availability of hand-wash facilities (OR 0.52; 95% CI 0.39–0.68; p < 0.001), availability of farm boots (OR 0.58; 95% CI 0.44–0.77; p < 0.001), burial disposal of dead bird (OR 0.67; 95% CI 0.51–0.89; p = 0.005), throw elsewhere for waste disposal of poultry farm (OR 0.35; 95% CI 0.23–0.53; p < 0.001) (Table 2).

**Table 2:** Univariable logistic regression analysis of risk factors for the presence of C. perfringens infections (n = 289) in broiler chicken farms (n = 25).

Variables	Category	Positive (%) (n = 289)	95% CI^a^	OR (95% CI)	p-value^b^
Farm location	Damietta (n = 566)	172(30.4)	26.62–34.16	1.56(1.19–2.04)	**0.001**^**b**^
	El-Dakhlia (n = 534)	117(21.9)	18.40–25.42	Reference	–
Housing system	Opened(n = 826)	215(26.03)	23.06–29.0	0.95(0.70–1.30)	0.75
	Closed(n = 274)	74(27.01)	21.75–32.27	Reference	–
Season	Summer (n = 308)	71(23.1)	18.33–27.77	1.79(1.16–2.77)	**0.009**^**b**^
	Fall (n = 272)	71(26.1)	20.88–31.33	2.11(1.36–3.28)	** < 0.001**^**b**^
	Winter (n = 262)	110(41.99)	36.01–47.96	4.32(2.82–6.62)	** < 0.001**^**b**^
	Spring (n = 258)	37(14.34)	8.97–19.71	Reference	–
History of coccidia infection	Yes (n = 714)	243(34.03)	30.80–37.26	3.81(2.70–5.38)	** < 0.001**^**b**^
	No (n = 386)	46(11.92)	8.69–15.15	Reference	–
Use of antimicrobial growth promoters	Yes(n = 542)	208(38.38)	34.29–42.47	3.67(2.74–4.91)	** < 0.001**^**b**^
	No (n = 558)	81(14.52)	11.59–17.44	Reference	–
Farm size^c^	Small(n = 390)	87(22.31)	18.20–26.42	0.72 (0.47–1.08)	0.11
	Medium(n = 164)	47(28.66)	21.73–35.59	Reference	–
	Large(n = 546)	155(28.39)	24.62–32.16	0.99(0.67–1.45)	0.95
Farm breed	Ross(n = 354)	132(37.29)	32.26–42.33	2.10 (1.49–2.95)	< 0.001^b^
	Sasso(n = 296)	61(20.6)	16.01–26.20	0.92(0.62–1.35)	0.66
	Cobb(n = 322)	71(22.05)	17.54–26.56	Reference	–
	Hubbard(n = 128)	25(19.53)	12.66–26.4	0.89(0.52–1.43)	0.56
Age of the birds^d^	Grower (n = 620)	217(35)	31.22–33.77	3.05(2.26–4.12)	** < 0.001**^**b**^
	Starter (n = 480)	72(15)	11.78–18.22	Reference	–
Litter type	Wet (n = 588)	220(37.41)	33.50–41.33	3.84(2.83–5.20)	** < 0.001**^**b**^
	Dry (n = 512)	69(13.48)	10.52–16.44	Reference	–
Disinfection of drinking water	Yes(n = 656)	101(15.4)	12.63–18.16	0.25(0.19–0.33)	** < 0.001**^**b**^
	No(n = 444)	188(42.34)	37.75–46.94	Reference	–
Feeder and drinker wash daily	Yes (n = 564)	90(15.96)	12.94–18.98	0.32(0.24–0.43)	** < 0.001**^**b**^
	No (n = 536)	199(37.13)	33.04–41.22	Reference	–
Hand-wash facilities available	Yes (n = 566)	114(20.14)	16.84–23.44)	0.52(0.39–0.68)	** < 0.001**^**b**^
	No (n = 534)	175(32.77)	28.79–46.75	Reference	–
Are farm boots available	Yes (n = 526)	110(20.91)	17.44–24.39	0.58(0.44–0.77)	** < 0.001**^**b**^
	No(n = 574)	179(31.18)	27.39–34.97	Reference	–
Disposal of dead bird	Burial (n = 449)	98(21.83)	18.01–25.65	0.67(0.51–0.89)	**0.005**^**b**^
	Throw elsewhere (n = 651)	191(29.34)	25.84–32.84	Reference	–
Waste disposal of poultry farm	Use as fertilizer (n = 881)	261(29.63)	26.61–32.65	Reference	–
	Throw elsewhere (n = 219)	28(12.79)	8.37–17.21	0.35(0.23–0.53)	** < 0.001**^**b**^

a-Confidence interval, b-significance, which is ≤ 0.05, c-farm size which are small (≤ 3000), medium (3000–5000) and large (> 5000), d -chicken age: starter (≤ 21 days), and grower (≥ 22 days).

Twelve risk factors were selected for the multivariable logistic regression analysis since the univariate analysis showed that they were statistically significant. The final model found several risk variables for *C. perfringens* infection at the farm level, which are listed in (Table 3). Significant correlations between *C. perfringens* infection and solely the age of the birds was found by multivariate analysis conducted at the farm level (Table 3). It was discovered that broiler farms with older age (grower; ≥ 22 days) had 2.09 times (95% CI 1.05–4.21, p = 0.037) greater odds ratio of *C. perfringens* infection.

**Table 3:** Multivariable logistic regression analysis of risk factors for the presence of C. perfringens infections (n = 289) in broiler chicken farms (n = 25).

Variables	Category	OR^∗^(95%CI)	p-value
Location	Damietta	0.95(0.57–1.60)	0.854
	El-Dakhlia	Ref	
Season	Summer	0.81(0.44–1.51)	0.510
	Fall	0.83(0.45–1.52)	0.537
	Winter	0.87(0.41–1.81)	0.700
	Spring	Ref	–
History of coccidia infection	Yes	0.71(0.33–1.52)	0.377
	No	Ref	–
Use of antimicrobial growth promoters	Yes	1.49(0.87–2.58)	0.150
	No	Ref	–
Age	Grower	2.09(1.05–4.21)	**0.037**
	Starter	Ref	–
Litter type	Wet	1.47(0.55–3.91)	0.443
	Dry	Ref	–
Disinfection of drinking water	Yes	0.72(0.30–1.74)	0.467
	No	Ref	–
Feeder and drinker wash daily	Yes	0.52(0.24–1.15)	0.108
	No	Ref	–
Hand-wash facilities available	Yes	0.93(0.28–3.11)	0.904
	No	Ref	–
Are farm boots available	Yes	1.15(0.26–5.19)	0.854
	No	Ref	–
Disposal of dead bird	Burial	0.91(0.32–2.61)	0.864
	Throw elsewhere	Ref	–
Waste disposal of poultry farm	Use as fertilizer	Ref	–
	Throw elsewhere	0.74(0.29–1.86)	0.528

95% CI 95% confidence interval, OR: Odds ratio, significant predictors in **bold.**

### Antibiotic resistance profiles

The antibiotic susceptibility results of 289 *C. perfringens* strains that were screened using the disk diffusion test for 28 appropriate antimicrobial drugs from 16 different antibiotic classes are shown in (Table 4). The greatest resistance of *C. perfringens* strains was noted in sulfamethoxazole-trimethoprim (94.5%), followed by erythromycin and imipenem (94.1%, for each), penicillin, ampicillin, streptomycin, and gentamycin (90.7%), ampicillin/sulbactam (89.9%), cefuroxime and cefepime (85.8%), nalidixic acid (85.1%), tetracycline (78.9%), ofloxacin, clindamycin, and colistin sulfate (70.6%), rifampicin (64.7%), amoxicillin, oxacillin, and ciprofloxacin (50.2%, for each), amoxicillin/clavulanic acid (48.4%), cefotaxime (46%), and amikacin (41.5%). By contrast, the resistance of *C. perfringens* strains was low to ceftriaxone, chloramphenicol (29.4%, for each), levofloxacin (28%), vancomycin and teicoplanin (21.8%, for each). Notably, every isolate that was tested shown meropenem susceptibility.

**Table 4:** Ecological distribution of antibiotic resistance of Clostridium perfringens (n = 289) in broiler chicken farms (n = 25).

Sample source		Sample type	Total no. of isolates	Antibiotics								
				SXT	E	IPM	p	AM	S	CN	SAM	CXM
Chickens’ clinical samples	Suspected live chicken	Cloacal swab	64	62	62	63	58	58	59	60	58	53
	Apparently healthy chicken	Feces	45	44	43	42	44	44	41	42	43	41
	Dead chicken	Intestine	82	79	79	79	77	77	78	77	77	75
		Liver	82	79	79	79	77	77	78	77	77	74
Total			273	264	263	263	256	256	256	256	255	243
Environmental samples		Litter	11	8	8	8	6	6	6	6	5	5
		Feed	3	1	1	1	0	0	0	0	0	0
		Wall swab	1	0	0	0	0	0	0	0	0	0
		Workers hand swab	1	0	0	0	0	0	0	0	0	0
Total			16	9	9	9	6	6	6	6	6	5
Overall total			289	273	272	272	262	262	262	262	260	248
Resistance %			100	94.5	94.1	94.1	90.7	90.7	90.7	90.7	89.9	85.8

Sample source		Sample type	Total no. of isolates	Antibiotics								
				CPM	NE	TE	OFX	DA	CT	RD	AX	OX
Chickens’ clinical samples	Suspected live chicken	Cloacal swab	64	51	52	45	40	38	40	36	28	28
	Apparently healthy chicken	Feces	45	43	41	38	37	39	37	37	28	28
	Dead chicken	Intestine	82	76	75	70	60	60	60	55	40	40
		Liver	82	73	74	71	63	63	63	57	47	47
Total			273	243	242	224	200	200	200	185	143	143
Environmental samples		Litter	11	5	4	4	4	4	4	2	2	2
		Feed	3	0	0	0	0	0	0	0	0	0
		Wall swab	1	0	0	0	0	0	0	0	0	0
		Workers hand swab	1	0	0	0	0	0	0	0	0	0
Total			16	5	4	4	4	4	4	2	2	2
Overall total			289	248	246	228	204	204	204	187	145	145
Resistance %			100	85.8	85.1	78.9	70.6	70.6	70.6	64.7	50.2	50.2

Sample source		Sample type	Total no. of isolates	Antibiotics								
				CIP	AMC	CTX	AK	CTR	C	LEV	VA	TEC
Chickens’ clinical samples	Suspected live chicken	Cloacal swab	64	26	25	23	21	14	14	12	12	12
	Apparently healthy chicken	Feces	45	30	27	25	24	18	18	17	14	14
	Dead chicken	Intestine	82	40	41	41	37	25	25	24	17	17
		Liver	82	47	47	44	38	28	28	28	20	20
Total			273	143	140	133	120	85	85	85	63	63
Environmental samples		Litter	11	2	0	0	0	0	0	0	0	0
		Feed	3	0	0	0	0	0	0	0	0	0
		Wall swab	1	0	0	0	0	0	0	0	0	0
		Workers hand swab	1	0	0	0	0	0	0	0	0	0
Total			16	2	0	0	0	0	0	0	0	0
Overall total			289	145	140	133	120	85	85	85	63	63
Resistance %			100	50.2	48.4	46	41.5	29.4	29.4	29.4	21.8	21.8

*SXT* sulphamethazole/trimethoprim, *E* erythromycin, *IPM* imipenem, *P* penicillin, *AM* ampicillin, *S* streptomycin, *CN* gentamicin, *SAM* ampicillin/sulbactam, *CXM* cefuroxime, *CPM* cefepime, *NA* nalidixic acid, *TE* tetracycline, *OFX* ofloxacin, *DA* clindamycin, *CT* colistin sulfate, *RD* rifampicin, *AX* amoxicillin, *OX* oxacillin, *CIP* ciprofloxacin, *AMC* amoxicillin/clavulanic acid, *CTX* cefotaxime, *AK* amikacin, *CTR* ceftriaxone, *C* chloramphenicol, *LEV* levofloxacin, *VA* vancomycin, *TEC* teicoplanin, *MEM* meropenem.

Based on sample type, the strains from the chickens’ clinical samples were highly resistance to sulfamethoxazole-trimethoprim (96.7%, 264/273) and erythromycin and imipenem (96.3%, 263/273, each), while the strains from the environmental samples showed low resistance to sulfamethoxazole-trimethoprim, erythromycin and imipenem (56.3%, 9/16, each).

*C. perfringens* strains showed nine multi-resistance patterns to antibiotics ranged from 9 to 22 (Table 5). The predominant multi-resistance pattern was SXT, E, IPM, P, AM, S, CN, SAM, CXM, CPM, NE, TE, OFX, DA, CT, RD, AX, OX, CIP, AMC, CTX, AK pattern (41.5%, 120/289). Among the evaluated *C. perfringens* isolates, 94.5% (273/289) were categorized as multidrug-resistant strains, meaning they could withstand three or more antibiotics. Additionally, the MAR index had an average of 0.58 and varied from 0.32 to 0.79 (Table 5).

**Table 5:** Antibiotic resistance pattern of multi-resistant *Clostridium perfringens* strains (n = 289) in broiler chicken farms.

Antibiotics pattern profile	Antibiotics	No. of resistance antibiotics	No. of isolates (%)	MARI (%)
1	SXT, E, IPM, P, AM, S,CN, SAM, CXM, CPM,NE, TE, OFX, DA, CT, RD, AX, OX, CIP, AMC, CTX, AK	22	120	0.79
2	SXT, E, IPM, P, AM, S,CN, SAM, CXM, CPM,NE, TE, OFX, DA, CT, AMC, CTR, C, LEV, VA, TEC	21	3	0.75
3	SXT, E, IPM, P, AM, S,CN, SAM, CXM, CPM,NE, TE, OFX, DA, CT, RD, CTR, C, LEV, VA, TEC	21	1	0.75
4	SXT, E, IPM, P, AM, S,CN, SAM, CXM, CPM,NE, TE, OFX, DA, CT, CTR, C, LEV	18	80	0.64
5	SXT, E, IPM, P, AM, S,CN, SAM, TE, RD, AX, OX, CIP, AMC, VA, TEC	16	14	0.57
6	SXT, E, IPM, P, AM, S,CN, SAM, CXM, CPM,NE, RD, VA, TEC	14	42	0.5
7	SXT, E, IPM, P, AM, S, CN, CXM, CPM, AMC, CTX, VA, TEC	13	2	0.46
8	SXT, RD, AX, OX, CIP, AMC, CTX, CTR, C, LEV, VA, TEC	12	1	0.43
9	SXT, E, IPM, TE, RD, AX, OX, CIP, CTX	9	10	0.32

*SXT* sulfamethoxazole/trimethoprim; *E* erythromycin, *IPM* imipenem, *P* penicillin; *AM* ampicillin, *S* streptomycin, *CN* gentamicin, *SAM* ampicillin/sulbactam, *CXM* cefuroxime, *CPM* cefepime, *NA* nalidixic acid, *TE* tetracycline, *OFX* ofloxacin, *DA* clindamycin, *CT* colistin sulfate, *RD* rifampicin, *AX* amoxicillin, *OX* oxacillin, *CIP* ciprofloxacin, *AMC* amoxicillin/clavulanic acid, *CTX* cefotaxime, *AK* amikacin, *CTR* ceftriaxone, *C* chloramphenicolm, *LEV* levofloxacin, *VA* vancomycin, *TEC* teicoplanin.

### Antibiotic resistant gene profiles

PCR assays were used to identify the presence of resistance genes in *C. perfringens* strains (n = 289). The following resistance genes were found to be highly prevalent: The resistance gene for aminoglycosides (*aph*A1) was present in 100% of *C. perfringens*. The carriage rates of β-lactam resistance genes *bla*_TEM_, *amp*C, *bla*_CTX_, *bla*_SHV_, and *bla*_Z_ were 85.8% (248/289), 50.2% (145/289), 46% (133/289), 35.6% (103/289), and 25.6% (74/289), respectively, with absence of *bla*_OXA-10_, *bla*_OXA-48_, *bla*_GES_, *bla*_IPM_, *bla*_VIM_, *bla*_KPC_, *bla*_NDM-1,_ *bla*_DHA_, *bla*_SFO_. The carriage rates of tetracycline resistance genes *tet*A, *tet*M, and *int-Tn*R were 78.9% (228/289), 42.2% (122/289), and 25.6% (74/289), respectively. The carriage rates of macrolide resistance genes *erm*B, *mef*A, and *msr*A were 85.1% (246/289), 50.2% (145/289), and 28.4% (82/289), respectively. Streptomycin resistance gene *aad*A was observed in 57.4% (166/289) isolates. Antibiotics referred to as quinolones, including *qnr*S, *qnr*D, *qnr*A, and *qnr*B had carriage rates of 85.1% (246/289), 60.2% (174/289), 50.2% (145/289), and 28% (81/289), respectively, while fluoroquinolone genes *gyr*A and *par*C were 15.6% (45/289). The carriage rates of s*ul*1 and *sul*2 (sulfonamide resistance genes) were 80.6% (233/289) and 60.2% (174/289), respectively. Meanwhile, trimethoprim resistance gene (*drf*A-1), chloramphenicol (*cat*A), and glycopeptides resistance gene (*van*A) were 75.1% (217/289), 57.4% (166/289), and 21.8% (63/289), respectively.

### Genetic diversity of *Clostridium perfringens* strains

The electrophoretic profile of DNA fragments gotten from 289 *C. perfringens* strains following ERIC-PCR typing produced 1–2 bands with sizes ranging from 100 to 800 bp. A visual examination of the banding patterns displayed 8 genotypes (A-H), with type D strains accounting for the highest proportion (42.56%, 123), followed by ERIC F (14.5%, 42), ERIC H (14.2%, 41), ERIC C (10%, 29), ERIC A (5.2%, 15), ERIC B (4.8%, 14), ERIC G (4.5%, 13), and ERIC E (4.2%, 12).

**Fig. 1:**
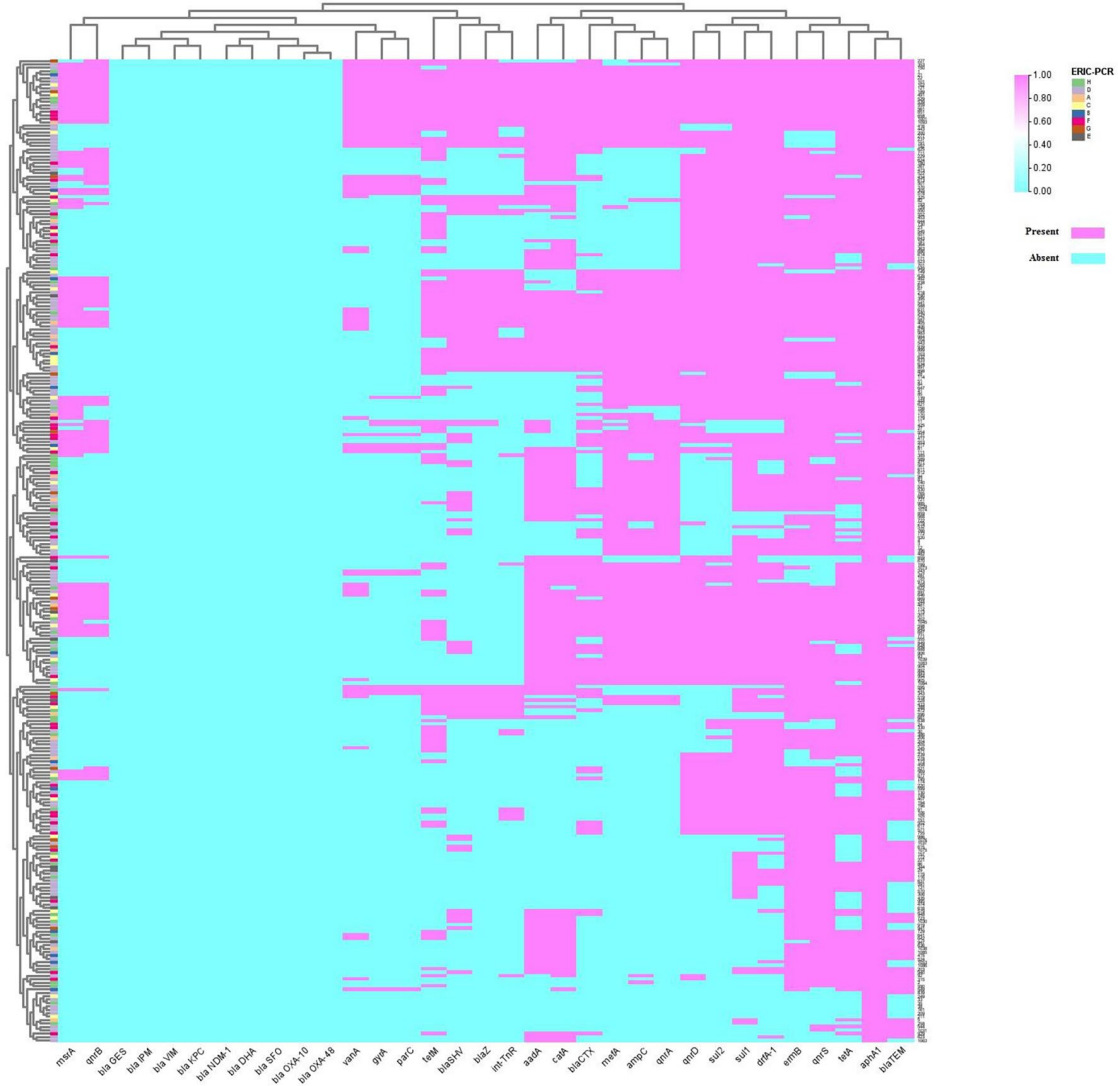
The heat map with hierarchical clustering analysis representing data on antibiotic resistant gene profiles and genetic diversity (ERIC‑PCR) of 289 *Clostridium perfringens* strains isolated from broiler farms.

The *C. perfringens* collected from 25 broiler farms were classified into distinct genotypes by sampling site and sample type, resulting in genetic diversity and heterogeneity, as seen by the ERIC-PCR dendrogram found as Supplementary Fig. S1 online. There were 8 ERIC types (A-H) for five farms (No. 1,3,5,13,15), while 5 ERIC types (D, E, F, G, and H) were common between four farms (No. 11,16,18,22). The predominant ERIC types were ERIC D among chickens’ clinical samples (liver, intestine 29.3%, 34/116; cloacal swabs 22.4%, 26/116, feces 18.9%, 22/116) followed by ERIC H (liver, intestine 32.5%, 13/40; cloacal swabs 25%, 10/40, feces 10%, 4/40) and F (cloacal swabs 29.7%, 11/37, liver, intestine 27%, 10/37; feces 10.8%, 4/37) than environmental samples. ERIC B was only common between cloacal swabs, intestines, and liver isolates, meanwhile ERIC D was common between isolates of litter (45.5%, 5/11). The heatmap with hierarchical clustering was applied to group samples based on antibiotic expression patterns, helping to identify genetic relationships and differences among samples (Fig. 1).

## Discussion

Numerous gastrointestinal and histotoxic illnesses in both humans and animals have often been linked to the infection induced by *C. perfringens*^19^. The most severe enteric bacterial disease in chicken, necrotic enteritis (NE), is caused by *C. perfringens* and causes significant financial losses for the poultry industry^3^. This is the first research study on risk factors associated with *C. perfringens* infection in Egyptian broiler farms. Overall, a total of 289 (26.3%) *C. perfringens* isolates from 1100 samples were identified with a higher prevalence in the intestine and liver samples (41%, each) which were collected from dead birds. Our results were in line with earlier reports from other countries, including China (23.4%)^20^, and Taiwan (29.6%)^21^. Several previous studies showed higher prevalence rates in Bangladesh (34.5%)^22^ and Egypt^23^ (55.9%). In contrast to other research conducted in the same country, Tresha et al.^24^, who sought to determine the prevalence of *C. perfringens* across various animal species and commercial broilers in India, found a significantly lower overall frequency of 10.3%, with a high occurrence in litter. Although the isolation levels of *C. perfringens* that have been discussed thus far may appear to be considerably different, multiple studies have demonstrated that the incidence of *C. perfringens* in poultry varies greatly between countries. The use of different detection techniques is thought to be the cause of these differences, and it has also been hypothesized that the host animal’s living conditions affect the prevalence rate of *C. perfringens*, even though it mostly colonizes the intestine^25^. This variation may also be caused by diverse terrains, changing seasons, varying climate conditions, and management techniques in various geographical areas.

The majority of *C. perfringens* isolates (78.5%) were identified as toxinotype A by toxinotyping results, which is in line with earlier studies indicating *C. perfringens* type A is mostly responsible for necrotic enteritis and the subclinical form of *C. perfringens* infection in poultry and the surrounding environment^23,26–28^. Our results revealed that 22.8% isolates were positive for *cpb2*, and 42.9% isolates harbored the *net*B gene identified as *C. perfringens* type G. These toxinotype G isolates showed the highest detection rate in samples from dead birds and were absent in environmental isolates. The absence of the *net*B gene in environmental *C. perfringens* isolates, in contrast to those from chickens, may explain the lower shedding and limited environmental survival of toxinotype G^29^. Accordingly, the chromosomally encoded alpha-toxin is produced by *C. perfringens* type A strains, whereas the two typing toxins, α-toxin and netB toxin, are encoded by *C. perfringens* type G strains, which cause NE in chickens. By producing toxins that cause NE, the plasmid-encoded genes *cpb2* and *net*B are believed to contribute to *C. perfringens’* pathogenicity. Numerous animals with enteric-related diseases caused by *C. perfringens* carry the beta2-toxin gene, *cpb*2^26^. Xiu et al.^27^ reported that the positive *cpb2* rate of in all isolates of *C. perfringens* was 30.85% by PCR from ducks. Moreover, production of toxin netB was closely associated with NE-inducing *C. perfringens* in chickens^11,30^. Profeta et al.^31^ detected 20.5% *net*B gene positive *C. perfringens* in Italian poultry flocks with the subclinical form of NE. The netB prevalence is typically higher in diseased birds and exists in both healthy and diseased broilers^32–34^. However, the virulence factor of the *net*B gene alone is insufficient to identify isolates that may cause NE. The fact that netB-negative isolates can spread illness in the field either alone or in conjunction with a larger microbial consortium is another feasible explanation^35^.

Risk factors for *C. perfringens* infection causing NE to have mostly been investigated using epidemiological surveys. Univariate analyses have identified several potential risk factors associated with the prevalence of *C. perfringens*. The results of univariate analysis indicate that *C. perfringens* infections were strongly associated with winter season, history of coccidiosis, use of antimicrobial growth promoters, grower age, and wet litter that had role in the epidemiology of the disease. Our study showed an association between geographical variation and elevated prevalence of *C. perfringens* infection as farms from Damietta had a higher risk of *C. perfringens* occurrence (OR 1.56, 95% CI 1.19–2.04; p = 0.001) compared to those from El-Dakhlia^22^. This could be due to the heavier rainfall and relatively lower humidity in Damietta. Since the samples were gathered throughout the year, it may be possible that the colder and wetter winter months contributed to the higher recovery of *C. perfringens* isolates. *C. perfringens* infections have been found to be more common in the early winter and during the cold season^28^. The winter months in the UK see the highest frequency of *C. perfringens* infections, whereas the summer months see a decrease in incidence^6^.

Wet litter and coccidiosis are two common enteric disorders in broilers, that can be thought of true risk factors for the disease^6^. Wet litter is a frequent phenomenon on UK broiler farms (56%)^36^ and reported in Bangladesh as an independent risk factor (OR 8.3, 95% CI 1.5–44.6; p = 0.007)^24^. In the current research, the infection rates of *C. perfringens* were significantly correlated with a history of coccidiosis on farms (OR 3.81, 95% CI 2.70–5.38; p =  < 0.001) in comparison to farms where coccidiosis was not demonstrated. A prior study discovered that the history of coccidia infection was significantly related with flock level *C. perfringens* infection status (OR 33.01, 95% CI 2.14–507.59, p = 0.01)^24^. Field observations have suggested that coccidial infections have a part in the prevalence of NE induced by *C. perfringens* because the damage that coccidia inflict on the intestinal epithelium produces a condition that facilitates the rapid proliferation and synthesis of toxins by *C. perfringens*^6^.

This work detected a higher risk of *C. perfringens* infection in farms that utilized antimicrobial growth promoters (OR 3.67, 95% CI 2.74–4.91; p < 0.001) compared to farms where growth promoters were not used. This finding is compatible with other study that indicated the use of preventive antibiotics in broiler farms was significantly associated with *C. perfringens* infection (OR 1.81, 95% CI 1.00–3.28; p = 0.03)^6^. Also, *C. perfringens* infection was lower in non-medicated broiler farms^28^. Our survey’s findings did not show that these antibiotics, as currently used in Egyptian broiler farms, had a significant protective effect. This might be regarded as the development of antimicrobial resistance among *C. perfringens* from the usage of antimicrobials as antibiotic growth promoters^37^. Additionally, the use of antibiotics may cause *C. perfringens* to overgrow in the intestinal tract and produce more toxins, which could set the stage for the development of necrotic enteritis associated with *C. perfringens*^38^.

According to our study, farms with grower chickens had a greater incidence of *C. perfringens* infection (OR 3.05, 95% CI: 2.26–4.12; p < 0.001) in contrast to starter chickens. This finding is in line with other research that showed that greater age was associated with a higher risk of infection than younger birds in Bangladesh and Egypt^24,39^.

The variables that did not exhibit any significant connections with the occurrence of infection in this investigation, with the exception of bird age, but are frequently implicated in other studies are equally illuminating as the risk factors that were retained in our final model. The only significant correlations found by multivariate analysis at the farm level were between *C. perfringens* infection and grower birds older than 21 days (OR 2.09; 95% CI 1.05–4.21; p = 0.037) as opposed to starter birds younger than 21 days. As the primary risk factor for *C. perfringens* infection, which primarily affects broilers between the ages of 2 and 6 weeks, when maternal antibodies start to disappear from the chicken’s circulation, this finding is corroborated by other researchers. These significant immune status alterations make the body more susceptible to infection and proliferation by *C. perfringens*^40^. Therefore, strict hygienic management procedures on the farm and the use of growth promotors other than antibiotics are therefore crucial for preserving broiler productivity and boosting production profitability.

Multidrug-resistant *C. perfringens* strains among reared animals such as poultry is a global issue that transcends geographical boundaries and can indiscriminately impact people from all socioeconomic classes^33,41,42^. In the present work, *C. perfringens* strains showed highest resistance to sulfamethoxazole-trimethoprim (94.5%), followed by erythromycin and imipenem (94.1%, for each), penicillin, ampicillin, streptomycin, and gentamycin (90.7%, for each), ampicillin/sulbactam (89.9%), cefuroxime and cefepime (85.8%, for each), nalidixic acid (85.1%), tetracycline (78.9%), ofloxacin, clindamycin, and colistin sulfate (70.6%, for each), rifampicin (64.7%), amoxicillin, oxacillin, and ciprofloxacin (50.2%, for each) but were highly susceptible to vancomycin and teicoplanin. All the isolates were susceptible to meropenem. Multiple resistance of *C. perfringens* strains to these categories of antibiotics namely sulfonamides, macrolides, carbapenems, β-lactam, aminoglycosides, cephalosporins, quinolones, and tetracycline are great concerns to public health. These findings confirmed the previous observations that stated variable resistance amongst *C. perfringens* strains to frequently used antibiotics in the treatment of clinical and veterinary infections^16,17,43^. Eid et al.^34^ observed high resistance of *C. perfringens* strains isolated from Egyptian broiler chicken farms to cephalexin, streptomycin, colistin sulfate, erythromycin, sulfamethoxazole-trimethoprim, gentamycin, and oxytetracycline. Additionally, the current investigation found concerning levels of multidrug resistance (94.5%). The predominance of multidrug-resistant strains of *C. perfringens* recovered from poultry has been referred to by a number of researchers^42,44^. Multidrug-resistant bacteria are thought to likely originate from the inappropriate and uncontrolled use of antibiotics in veterinary care and on farms^45^. As a result, it is necessary to continuously monitor the development of antibiotic resistance and identify substitute approaches for the management and treatment of animal illnesses. Also, all *C. perfringens* strains isolated from the broiler farms in the current study had MAR index values greater than 0.20, suggesting that they came from high-risk sources where they had been exposed to antibiotics on a regular basis and so posed a significant risk^46^.

The potential spread of antimicrobial resistance genes to other pathogens and the abuse of broad-spectrum antibiotics may be reduced with a better understanding of *C. perfringens* resistance mechanisms. To find out if every multidrug resistant strain of *C. perfringens* in this study has at least one antimicrobial resistance gene, the mechanisms granting resistance to different classes of antibiotics were investigated. Numerous homologous genes have been discovered and characterized in various gram-negative bacteria that exhibit homologous combination, along with their mechanisms of resistance to antibiotics^47^. Antimicrobial resistance gene profiling among the recovered isolates revealed a higher presence of the aminoglycoside resistance gene *aph*A1 (100%) followed by *bla*_TEM_ (85.8%), *erm*B, *qnr*S (85.1%), *sul*1 (80.6%), *tet*A(78.9%), *drf*A-1 (75.1%), *qnr*D, *sul*2 (60.2%), *cat*A (57.4%), *aad*A (57.4%), *amp*C, *mef*A, *qnr*A (50.2%), *bla*_CTX_ (46%), and *tet*M (42.2%). These are mostly regular drug resistance genes in *C. perfringens*. The *aph*A1 determinants of the kanamycin and neomycin resistance and their configuration and location in the chromosome were established^48^. Lower carriage rates of aminoglycoside and macrolides resistance genes in *C. perfringens* were previously detected in China^43^. The prevalence of genes encoding β-lactamases (*bla*_TEM_ and *bla*_CTX_) in *C. perfringens* raised serious concerns for public health because these genes can be released into the environment and spread to other microflora, leading to the development of superbugs in the future, as was shown in Bangladish^49^. The most common macrolide resistance gene in *C. perfringens* was discovered to be the *erm*B gene^16^. The *bla* and *erm*B genes which were responsible for resistant isolates to beta lactam and erythromycin were detected in all the Egyptian tested clostridial strains^41^. However, lower percentages of *erm*B gene were found in *C. perfringens* from USA (0.8%) and Egypt (25%)^50,51^. The high detection rate of quinolone resistance gene (*qnr*D) (70.6%) was found^18^. Sulfonamide resistance genes (*sul*1 & *sul*2) were commonly detected among sulfonamide resistance isolates of *C. perfringens* in China and Egypt^18,52^. *C. perfringens* strains were found to possess tetracycline-resistant genes, *tet*A(P) and *tet*B(P), which are known to encode proteins that facilitate active tetracycline efflux and ribosomal protection tetracycline-resistant mechanisms^53^. Various tetracycline resistance genes (*tet*A, *tet*B, and *tet*44) were detected in 16/20 *C. perfringens* isolates^54^. Tetracycline resistance levels and the frequency of the tetracycline-resistance genes (*tet*A(P), *tet*B(P), and *tet*(M)) were variable among *C. perfringens* isolates from various sources^55^.

The chloramphenicol-resistant strain of *C. perfringens* has been shown to be able to transfer the protein encoded by the *cat*P resistance gene on Tn4453 transposon through plasmid conjugation and inactivate chloramphenicol when exposed to antibiotic stress^56^. One mechanism of AmpC β-lactamase, class C enzymes under the Ambler classification scheme, resistance is inducible resistance via chromosomally encoded *amp*C genes that are mostly detected in the resistant Gram-negative bacteria to penicillin and extended-spectrum cephalosporins^57^. Moreover, *gyr*A was also discovered in the genome of *C. perfringens* IRMC2505A by the use of k-mer-based detection techniques for antimicrobial resistance genes^53^.

The existence of antimicrobial resistance genes is significant as most antimicrobial agents are extensively used in both human and animal infections as prophylaxis and therapy potentially selecting for drug resistance. The continuous misuse of these antibiotics has facilitated the emergence of microbial resistance which has limited the antimicrobial effectiveness. Resistance can occur due to mutations of the targeted site or can be acquired through horizontal gene transfer of extrachromosomal elements such as insertion sequences, phages, and plasmids amongst *C. perfringens* isolates in the environment^49^.

ERIC is the best practical way for screening a large number of samples in molecular epidemiology research, when cost-effective, straightforward, and less time-consuming methods are needed. Bacterial genotyping and genetic connections have been extensively studied using the ERIC-PCR technology^18^. To our knowledge, little is known about the molecular type of *C. perfringens* isolated from Egyptian broiler farms using ERIC-PCR and from various Egyptian geographical locations. The purpose of our study was to use ERIC-PCR to evaluate the genetic relatedness of *C. perfringens* type A and type G isolates that were successfully recovered from broiler farms in order to explain the diversity in *C. perfringens* subtypes in broiler farms. In the current research, the ERIC-PCR analysis demonstrated that the *C. perfringens* originated from twenty-five broiler farms were genetically diverse and heterogeneous. The heterogeneity was supposed to be as the strains originated from various types of samples (chickens’ clinical and environmental samples) and sampling locations. Among them, ERIC D genotype is the main epidemic type circulating in broilers. In previous study, six genotypes (I-VI) of *C. perfringens* strains from ruminants identified by ERIC-PCR which confirmed that different genotypes existed in different regions in Xinjiang, China, showing obvious genetic diversity^18^. Our study showed low similarity between the broiler farms in *C. perfringens* genotypes which may be attributed to the geographical location of farms in the same country (Egypt). Furthermore, it was found that strains with the same genotype were diverse in their antibiotic resistance genes, and toxin genes. For example, strains (n = 1&2), which had a similar ERIC H type, were different in their antibiotic resistance genes, and toxin genes.

## Conclusion

The study highlights the prevalence, toxinotyping, and multi-drug resistance of *C. perfringens*, which displays obvious genetic diversity along with the risk factors of infection in broiler farms in the selected areas of Egypt. The data confirms that Type A is the most prevalent amongst *C. perfringens* isolates and highlights the role of netB toxin in producing necrotic enteritis in broiler chickens. Location, season, history of coccidia infection, use of antimicrobial growth promoters, birds age, litter type, and biosecurity strategy were correlated to the occurrence of *C. perfringens* infection. The work results could lay a basis for farm-level intervention strategies to prevent *C. perfringens* infection in commercial broiler farms. Policies addressing such risk factors could lead to the efficiency of control and preventative measures in lowering the risk of *C. perfringens* infection on broiler farms. Understanding how antibiotic resistance works also aids in the development of novel treatments for infectious diseases, which are desperately required.

## Material and methods

### Ethics

The Animal Research Ethical Committee of the Faculty of Veterinary Medicine at Mansoura University in Egypt granted ethical approval for the collection of samples and clinical data from broiler farms (Code number VM. R. 24.11.197). All methods were carried out in accordance with relevant guidelines and regulations. Also, the study was conducted following ARRIVE guidelines. Before enrollment, the owner’s verbal consent to participate in the study was acquired.

### Study areas and farms

**Fig. 2:**
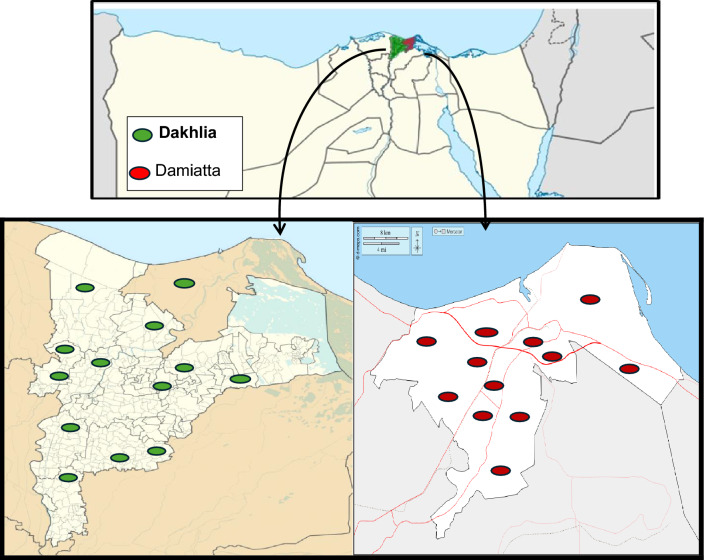
Locations of the surveyed broiler farms (n = 25) with a flock size ≥ 2000 birds in two districts (El-Dakhlia and Damiatta Governorates) located in Northern Coast of Egypt indicated by colored spots on the map. Figure was created by photoshop Version 23.5.2023.

A cross-sectional investigation was conducted to investigate the prevalence of *C. perfringens* causing necrotic enteritis and the associated risk factors in the environment of broiler farms across two distinct regions (El-Dakhlia and Damietta Governorates), located in Northern Coast of Egypt (Fig. 2). The research was conducted over a long period of time, from March 2023 to February 2024. The region has a moderate climate, which is defined by hot, dry summers and mild winters with little rain falling along the coast. The average yearly temperature was between 18 °C and 31 °C, with a lower humidity of between 63 and 70%. This study included twenty-five broiler farms (13 from El-Dakhlia and 12 from Damietta Governorates), with a farm size ranging from 2,000–50,000 individuals, aged between 13–35 days old, and reared in litter under open and closed systems of housing. Rice husks or wood shavings were used as bedding materials. The choice of broiler farms was based on their owners’ willingness to permit sample collection and participate in such a study across these two distinct regions.

### Questionnaire

A pretested semi-structured questionnaire was used to gather data from farm managers during the sampling process. The veterinarians (if available) from participating farms assisted in the formulation of the questionnaire. The questionnaire for broiler farmers included fifteen closed-ended questions. The survey collected detailed data on bird-related factors (e.g., age, farm size, and farm breed), along with biosecurity-related parameters (e.g., litter type, disinfection of drinking water, feeder and drinker wash daily, hand-wash facilities available, farm boots available, disposal of dead birds, and waste disposal of poultry farm), and general information on the farm (e.g., farm location, season, housing system, history of coccidia infection, and use of antimicrobial growth promoters). Farmers didn’t alter litter during rearing. To make the questionnaire’s content easier for farmers to understand, it was translated into a local language.

All methods were carried out in accordance with relevant guidelines and regulations. All experimental protocols were approved by the Animal Research Ethical Committee of the Faculty of Veterinary Medicine at Mansoura University, Egypt (approval no. VM. R. 24.11.197). Informed consent was obtained from all subjects and/or their legal guardian(s).

### Sampling

A total of 1100 samples including of cloacal swabs from suspected live birds with necrotic enteritis (n = 250; 10 samples per farm), intestinal scrapings (n = 200), liver (n = 200) from dead birds with symptoms of necrotic enteritis, feces from apparently healthy birds (n = 250; 10 samples per farm), and the environmental samples (n = 200) (litter, water, feed, feeder, drinker, wall, fans, and workers hand swabs; n = 25 for each type; one pooled sample per farm) from twenty-five broiler farms. The number of dead birds sampled per flock for intestinal and liver tissues was as follows: 9 birds from 1 flock, 5 birds from each of 2 flocks, 7 birds from each of 3 flocks, 6 birds from each of 5 flocks, 8 birds from each of 5 flocks, and 10 birds from each of 9 flocks. The birds exhibited symptoms of necrotic enteritis either as acute or chronic clinical forms. Acute form showed high mortality, depression, decreased appetite, ruffled feathers, and diarrhea, while chronic cases showed diarrhea (foamy and foamy pings with a definite zone of fluidity), dehydration, emaciation, unwillingness to move and reduced growth. Samples (intestine and liver) were gathered from recently dead birds as soon as possible and a post-mortem inspection was performed. The intestine contained gas bubbles, thick, foul-smelling, and brownish watery exudate. After being gathered in sterile plastic bags, the samples were promptly taken to the laboratory for analysis in an ice box.

### Bacteriological analysis

The samples were preliminary enriched in the Robertson’s Cooked Meat media (RCM broth) (HiMedia, Maharashtra, India) followed by anaerobic incubation at 37 °C for 24 h in anaerobic Gas pack jar with AnaeroPack-Anaero Kits (Mitsubishi Gas Chemical Inc., Japan). A loopful of enrichment broth was streaked on 10% sheep blood agar (Himedia, Maharashtra, India) supplemented with 200 μg/ml neomycin sulfate and anaerobically incubated at 37 °C for 24 h. To isolate pure colonies, subcultures were streaked onto Tryptose Sulphite Cycloserine agar (TSC; HiMedia, Maharashtra, India) supplemented with 400 mg/L D-cycloserine (HiMedia, Maharashtra, India) and anaerobically incubated at 37 °C overnight. Colonies displaying *C. perfringens* were successively assessed and documented simultaneously. The standard technique was followed for the Gram’s staining, motility test, and biochemical testing (catalase, urease, sugar fermentation, gelatin liquefaction, nitrate reduction, and lecithinase response tests)^58^. For further analysis, pure isolates exhibiting standard characteristics were maintained in Brain Heart Infusion broth (BHI) (Himedia, India) with 24% glycerol at -80 °C.

### Molecular toxinotyping of *C. perfringens*

The bacterial DNA was extracted from overnight cultures on 5% sheep blood agar by boiling method^59^. In short, 200 µL of distilled water was used to make a suspension of five colonies from overnight cultures on BHI agar. The colonies were then boiled for 10 min, cooled on ice, and centrifuged at 16,000 × g for 10 min to get rid of any debris. In the PCR tests, the supernatant served as the template DNA. The alpha (*cpa*) toxin gene, which is found in all *C. perfringens* toxinotypes, was tested using single PCR^32^. Using published primers and methods, isolates that tested positive for the *cpa* gene were examined for the beta (*cpb*), beta2 (*cpb2*), epsilon-toxin (*etx*), iota-toxin (*iap*), *cpe*, *tpe*L, and *net*B-toxin genes using standard PCR tests in an Applied Biosystem 2720 Thermal Cycler (USA) **(**see Supplementary Table S1 online**)**. One µL of individual primer (Metabion, Germany), five µL of DNA template, 5.5 µL of PCR-grade water, and 12.5 µL of 2 × PCR master mix (Promega, Madison, USA) made up the 25 µL PCR reactions. Amplicons were separated on a 1.5% agarose gel (Sigma, USA) and photographed using an UV illumination. In this study, DNA of *C. perfringens* type A strain kindly supplied as a reference strain from Anaerobic Unit in the Animal Health Research Institute at Dokki, Cairo, Egypt, while sterile saline served as a negative control.

### In-vitro antimicrobial susceptibility testing

Susceptibility of *C. perfringens* isolates to twenty-eight commercially available antimicrobial agents were examined by a disc diffusion method in accordance with Clinical and laboratory standards institute (CLSI)^60^ on Muller Hinton agar plate (Oxoid, UK). The following antibiotic discs (Oxoid, UK) were commonly utilized in human and poultry farms; Penicillins [penicillin G (P; 10 IU), ampicillin (AM; 10 µg)], β-lactam [amoxicillin (AX; 10 µg), oxacillin (OX; 1 µg), amoxicillin/clavulanic acid (AMC; 20/10 µg), ampicillin/sulbactam (SAM; 20 µg)], cephalosporines [cefuroxime (CXM; 30 µg), ceftriaxone (CTR; 30 µg), cefotaxime (CTX; 30 µg), cefepime (CPM; 30 µg)], carbapenems [imipenem (IPM; 10 µg), meropenem (MEM; 10 µg)], aminoglycosides [gentamycin (CN; 10 µg), streptomycin (S; 10 µg), amikacin(AK; 30 µg) ], macrolides [erythromycin (E; 15 µg)], quinolones [nalidixic acid (NA; 30 µg)], fuoroquinolones [ciprofloxacin (CIP; 5 µg), ofloxacin (OFX; 5 µg), levofloxacin (LEV; 5 µg)], sulfonamides [sulfamethoxazole-trimethoprim (SXT; 23.75/1.25 μg)], amphenicols [chloramphenicol (C; 30 µg)], lincosamides [clindamycin (DA; 2 µg], tetracyclines [tetracycline (TE; 30 µg)], glycopeptides [vancomycin (VA; 30 µg), lipopeptides [colistin sulfate (CT; 10 µg)] and lipoglycopeptides [teicoplanin (TEC; 30 µg)], and rifamycin [rifampicin (RD; 5 µg)]. In brief, an overnight Brain Heart Infusion broth (BHI; Microexpress, Accumix, Ltd, India) was diluted with saline to an optical density equal to 0.5 McFarland standards. An inoculation was then made onto Mueller Hinton agar medium, and antimicrobial discs were placed on top. In a Gas Pack anaerobic jar, plates were incubated anaerobically for 24 h at 37 °C. The European Committee on Antimicrobial Susceptibility Testing (EUCAST) and CLSI were used to assess and evaluate the inhibition zone’s diameter^61^. The *C. perfringens* strain ATCC 19574 was employed as quality control. The ratio of the number of antibiotics to which *C. perfringens* isolates showed resistance to the number of antibiotics for which the isolates were tested was known as the multiple antibiotic resistance index, or MARI. When an isolate is resistant to at least one agent from three or more antibiotic classes, it is said to be multidrug resistant (MDR)^62^.

### Characterization of antibiotic-resistant genes

The PCR profiling of *C. perfringens* strains’ antibiotic resistance (AR) determinants included screening 32 antibiotic-resistant genes that encoded relevant β-lactamases (*amp*C, *bla*_TEM_, *bla*_SHV_, *bla*_SFO-1_, *bla*_DHA-1_, *bla*_CTX_, *bla*_OXA-10_, *bla*_OXA-48_, *bla*_GES_, *bla*_IPM_, *bla*_VIM_, *bla*_KPC_, *bla*_NDM-1,_ *bla*_Z)_, aminoglycosides (*aph*A1), Streptomycin (*aad*A), macrolides (*erm*B, *msr*A, *mef*A), quinolones (*qnr*A, *qnr*B, *qnr*S, *qnr*D), fluoroquinolones (*gyr*A, *par*C), tetracyclines (*int*-TnR, *tet*A, *tet*M), sulfonamide (*sul*1, *sul*2), trimethoprim (*drf*A-1), glycopeptides (*van*A), and chloramphenicol (*cat*A) by specific primers and amplification conditions **(**see Supplementary Table S2 online**)** and following the method previously described above.

### Molecular typing by ERIC-PCR method

*C. perfringens* was ERIC-PCR typed to determine the genetic relatedness between isolates as previously reported using the primers ERIC (see Supplementary Table S1 online)^18^. In summary, a 25 μL PCR reaction is made up of 6 μL of DNA template, 4.5 μL of PCR-grade water, 1 μL of each primer (Metabion, Germany), and 12.5 μL of 2 × PCR master mix (Promega, Madison, USA). The following thermal cycles (Biometra) were used for the amplification: pre-denaturation at 94 °C for 5 min, 35 cycles (denaturation at 94 °C for 30 s, annealing at 52 °C for 1 min, and extension at 72 °C for 1 min), and final extension at 72 °C for 12 min. Gel electrophoresis photo-documented under UV illumination (Alpha Innotech) was used to assess the electrophoretic bands of the PCR generated products, and the data was arranged. Based on whether each band was present or absent, the ERIC fingerprinting data was transformed into a binary code. Ward’s hierarchical clustering procedure and the unweighted pair group approach with arithmetic average (UPGMA) were used to produce the dendrogram. SPSS version 22 was used to demonstrate cluster analysis and dendrogram creation (IBM, Armonk, NY, USA, 2013). An online tool (https://planetcalc.com/1664/) was used to examine the similarity index (Jaccard/Tanimoto coefficient and number of intersecting elements) among all samples.

### Statistical analysis

Microsoft Excel 2010 spreadsheets were used to record the data from the interviews and laboratory tests. IBM SPSS Statistics 27.0 software (SPSS Inc., http://www.spss.com.hk) was used for all statistical analyses. The questionnaires yielded descriptive data, including parameters linked to biosecurity, bird-related factors, and general information. A 95% confidence interval (CI) for the prevalence of *C. perfringens* infections was first computed. Chi-square test was done to determine the relationship between the prevalence of *C. perfringens* infections (outcome variable) and predictor variables (risk factors) including bird-related factors, biosecurity-related parameters and general information. A univariate logistic regression model was used to estimate the association between participant characteristics (risk variables) and *C. perfringens* positive status. The odds ratio (OR) values with a 95% CI were computed, and p < 0.05 was supposed to be statistically significant. Inclusion in the multivariable logistic regression model was based on the variables that had a p-value of less than 0.05 in the univariable analysis. A p-value of less than 0.05 was deemed statistically significant. Lastly, TBtools was used to create a representative heat map based on antibiotic resistance genes and genetic linkages found in isolates from broiler farms using ERIC-PCR.

## Supplementary Information

Supplementary Information.

## Data Availability

Data generated in this study are included in this published article.

## Abbreviations

NE: Necrotic enteritis
NetB: Necrotic enteritis B-like toxin
PLC: Phospholipase C
ERIC-PCR: Enterobacterial repetitive intergenic consensus sequence-based PCR
RCM: Robertson’s cooked meat media
TSC: Tryptose sulphite cycloserine agar
BHI: Brain heart infusion broth
*cpa*: Alpha toxin gene
*cpb*: Beta toxin gene
*cpb2*: Beta2 toxin gene
*etx*: Epsilon-toxin gene
*iap*: Iota-toxin gene
CLSI: Clinical and laboratory standards institute
P: Penicillin
AM: Ampicillin
AX: Amoxicillin
OX: Oxacillin
AMC: Amoxicillin/clavulanic acid
SAM: Ampicillin/sulbactam
CXM: Cefuroxime
CTR: Ceftriaxone
CTX: Cefotaxime
CPM: Cefepime
IPM: Imipenem
MEM: Meropenem
CN: Gentamycin
S: Streptomycin
AK: Amikacin
E: Erythromycin
NA: Nalidixic acid
CIP: Ciprofloxacin
OFX: Ofloxacin
LEV: Levofloxacin
SXT: Sulfamethoxazole-trimethoprim
C: Chloramphenicol
DA: Clindamycin
TE: Tetracycline
VA: Vancomycin
CT: Colistin sulfate
TEC: Teicoplanin
RD: Rifampicin
EUCAST: The European committee on antimicrobial susceptibility testing
MARI: Multiple antibiotic resistance index
MDR: Multidrug resistant
CI: Confidence interval
OR: Odds ratio
