# Supplementary material for: Epidemiology of potential source, risk attribution of Clostridium perfringens from Egyptian broiler farms and genetic diversity of multidrug resistance strains
Source: Sci Rep. 2025 Aug 5;15:28638. doi: 10.1038/s41598-025-12519-0 (PMC12326005; doi:10.1038/s41598-025-12519-0)
Supplement: Supplementary file 1 — Supplementary Information. [file 41598_2025_12519_MOESM1_ESM.docx]

**Supplementary Table S1. Oligonucleotide primer sequences of toxin genes and amplified PCR product sizes used for *Clostridium perfringens* in this study.**

| **Target Gene** | **Primer** | **Primer Sequence (5’-3)** | **Annealing** | **PCR product size (bp)** | **Reference** |
| --- | --- | --- | --- | --- | --- |
| *cpa* | CPAlphaF  CPAlphaR | F: GCTAATGTTACTGCCGTTGA  R: CCTCTGATACATCGTGTAAG | 53 | 324 | Park et al.^1^ |
| *cpb2* | CPB2L  CPB2R | F:AGATTTTAAATATGATCCTAACC  R:CAATACCCTTCACCAAATACTC | 55 | 567 | Yang et al.^2^ |
| *cpb* | CPBL  CPBR | F:TCCTTTCTTGAGGGAGGATAAA  R:TGAACCTCCTATTTTGTATCCCA | 48 | 611 | Fan et al.^3^ |
| *etx* | CPETXL  CPETXR | F:TGGGAACTTCGATACAAGCA  R:TTAACTCATCTCCCATAACTGCAC | 55 | 396 |  |
| *iap* | CPIL  CPIR | F:AAACGCATTAAAGCTCACACC  R:CTGCATAACCTGGAATGGCT | 55 | 293 |  |
| *cpe* | CPEL  CPER | F:GGGGAACCCTCAGTAGTTTCA  R:ACCAGCTGGATTTGAGTTTAATG | 55 | 506 |  |
| *tpe*L | TPELF  TPELR | F:ATATAGAGGCAAGCAGTGGAG  R:GGAATACCACTTGATATACCTG | 50 | 466 | Coursodon et al.^4^ |
| *net*B | NETB | F:GGAAGGCAACTTTAAGTGGAAC  R:GTTTGTTCCTCGCCATTGAGT | 58 | 680 | Wu et al.^5^ |
| *ERIC* | ERIC1: ATGTAAGCTCCTGGGGATTCAC  ERIC2: AAGTAAGTGACTGGGGTGAGCG | | 52 | Xiaoting et al.^6^ | |

1. Fan, Y.C., Wang, C. L., Wang, C., Chen, T. C., Chou, C. H., Chou, C. H. & Tsai, H. J. Incidence and antimicrobial susceptibility to *Clostridium perfringens* in premarket broilers in Taiwan. *Avian Dis.* **60**(2), 444-449 (2016).

2. Yang, W. Y., Chou, C. H. & Wang, C. Characterization of toxin genes and quantitative analysis of netB in necrotic enteritis (NE)-producing and non-NE-producing *Clostridium perfringens* isolated from chickens. *Anaerobe* **54,** 115-120 (2018).

3. Park, J. Y. et al. Characterization of *Clostridium perfringens* isolates obtained from 2010 to 2012 from chickens with necrotic enteritis in Korea. *Poult. Sci.* **94,** 1158–1164 (2015).

4. Coursodon, C. F., Glock, R. D., Moore, K. L., Cooper, K. K. & Songer, J. G. TpeL-producing strains of *Clostridium perfringens* type A are highly virulent for broiler chicks. *Anaerobe* **18**(1), 117-121 (2012).

5. Wu, D. et al. Antimicrobial susceptibility and multilocus sequence typing of *Clostridium perfringens* isolated from yaks in Qinghai-Tibet plateau, China. *Front. Vet. Sci.* **9,** 1562 (2022).

6. Xiaoting, W. et al. Antimicrobial resistance profiling and molecular typing of ruminant-borne isolates of *Clostridium perfringens* from Xinjiang, China. *J. Glob. Antimicrob. Resist.* **27,** 41-45 (2021).

**Supplementary Table S2. Target antibiotic resistance genes, Primers sequences, amplicon sizes used for *Clostridium perfringens* in this study.**

| **Antimicrobial Class** | **Target gene** | **Primers sequences** | **Annealing** | **Amplified segment (bp)** | **Reference** |
| --- | --- | --- | --- | --- | --- |
| [**β-lactam**](https://en.wikipedia.org/wiki/Beta-lactam_antibiotic) | *amp*C | F: GATCGTTCTGCCGCTGTG  R: GGGCAGCAAATGTGGAGCAA | 56 | 271 | Oliver et al.7 |
|  | *bla*_TEM_ | F: ATGAGTATTCAACATTTTCGTG  R: TTACCAATGCTTAATCAGTGAG | 57 | 861 | Mathlouthi et al.8 |
|  | *bla*_SHV_ | F: CACTCAAGGATGTATTGTG  R: TTAGCGTTGCCAGTGCTCG | 56 | 822 | Pitout et al.^9^ |
|  | *bla*_CTX_ | F:SCSATGTGCAGYACCAGTAA  R ACCAGAAYVAGCGGBGC- | 55 | 585 | Ojdana et al.^10^ (2014) |
|  | *bla*_OXA-10_ | F:TATCGCGTGTCTTTCGAGTA  R: TTAGCCACCAATGATGCCC | 55 | 760 | Mirsalehian et al.^11^ 2010 |
|  | *bla*_OXA-48_ | F: GCTTGACCCTCGATT  R:GATTTGCTCCGTGGCCGAAA | 60 | 281 | Dallenne et al.^12^ (2010) |
|  | *bla*_IPM_ | F: TTGACACTCCATTTACDG  R: GATYGAGAATTAAGCCACYCT | 55 | 139 |  |
|  | *bla*_KPC_ | F: CATTCAAGGGCTTTCTTGCTGC  R: ACGACGGCATAGTCATTTGC | 55 | 538 |  |
|  | blaGES | F: AGTCGGCTAGACCGGAAAG  R:TTTGTCCGTGCTCAGGAT | 57 | 399 | Hou et al.^13^ |
|  | *bla*_SFO-1_ | F 5′-ATTCAGCAGCAACTGTCCG-3′  R 5′-ACGCTTATCGCTGGGAAT-3′ | 54 | 447 | Muratani et al.^14^ (2006) |
|  | *bla*_DHA_-1 | F: CCAGAATCACAATCGCCACC  R: TATCAGCAGTGGCAGCCGT | 64 | 405 | Guo et al.^15^ |
|  | *bla*_VIM_ | F:GATGGTGTTTGGTCGCATA  R: CGAATGCGCAGCACCAG | 60 | 390 | Sjolander et al.^16^ |
|  | *bla*_NDM-1_ | F: CAATATTATGCACCCGGTCG  R: ATCATGCTGGCCTTGGGGAA | 56 | 726 | Kim et al.^17^ |
|  | *bla*_Z_ | F: TACAACTGTAATATCGGAGG  R: CATTACACTCTTGGCGGTTTC | 58 | 861 | Rosato et al.^18^ |
| **Aminoglycosides** | *aph*A1 | F: ATGGGCTCGCGATAATGTC  R: CTCACCGAGGCAGTTCCAT | 55 | 600 | Maynard et al.^19^ |
| **Tetracyclines** | *tet*(A) | F: GGTTCACTCGAACGACGTCA  R: CTGTCCGACAAGTTGCATGA | 577 | 55 | Randall et al.^20^ |
|  | *tet*M | F: GTGGACAAAGGTACAACGAG  R :CGGTAAAGTTCGTCACACAC | 405 | 55 | Morvan et al. ^21^ |
|  | *Int*-Tn | F: GATGGTATTGATGTTGTAGG  R: GGTCTATATATTGACAAGACCG | 525 | 55 |  |
| **Macrolide** | *erm*B | F: GAAAAGGTACTCAACCAAATA  R :AGTAACGGTACTTAAATTGTTTAC | 636 | 55 |  |
|  | *msr(A)* | F: GCAAATGGTGTAGGTAAGACAACT  R ATCATGTGATGTAAACAAAAT | 401 | 55 |  |
|  | *mef(A)* | F AGTATCATTAATCACTAGTGC 345  R TTCTTCTGGTACTAAAAGTGG | 345 | 55 |  |
| **Streptomycin** | *aad* A1 | F: TATCCAGCTAAGCGCGAACT  R: ATTTGCCGACTACCTTGGTC | 56 | 447 | Odeyemi et al.^22^ |
| **Quinolones** | *qnr*A | F: GGGTATGGATATTATTGATAAA  R: CTAATCCGGCAGCACTATTA | 55 | 657 | López et al.^23^ |
|  | *qnr*B | F: GGMATHGAAATTCGCCACTG  R: TTTGCYGYYCGCCAGTCGAA | 55 | 263 |  |
|  | *qnr*S | F: AGTGATCTCACCTTCACCGC  R: CAGGCTGCAATTTTGATACC | 55 | 552 |  |
|  | *qnr*D | F:CGAGATCAATTTACGGGGAATA  R: AACAAGCTGAAGCGCCTG | 54 | 582 | Cavaco et al. ^24^ |
| **Fluoroquinolones** | *gyr*A | F: AGTGTAATTGTTGCCCG  R: ATATCGCCATCAACCGA | 470 | 55 | Godreuil et al.^25^ |
|  | *par*C | F: GAACGTGCGCTTCCAGA  R: GTTGCATAACCAGCGGA | 449 | 55 |  |
| **Sulfonamide** | *Sul1* | F: TTCGGCATTCTGAATCTCAC  R: ATGATCTAACCCTCGGTCTC | 55 | 822 | Maynard et al.^19^ |
|  | *Sul*2 | F: CGGCATCGTCAACATAACC  R: GTGTGCGGATGAAGTCAG | 55 | 722 |  |
| **Trimethoprim** | *drf*A1 | F: GGAGTGCCAAAGGTGAACAGC  R: GAGGCGAAGTCTTGGGTAAAAAC | 367 | 55 | Toro et al.^26^ |
| **Glycopeptides** | *van*A | F: ATTGCTATTCAGCTGTACTC  R: GGCTCGAGTTCCTGATGAAT | 55 | 559 | Mirzaie e t al.^27^ |
| **Chloramphenicol** | *cat*A1 | F: AGTTGCTCAATGTACCTATAACC  R: TTGTAATTCATTAAGCATTCTGCC | 547 | 55 | Van e t al.^28^ |

7. Oliver, A. et al. Mechanisms of decreased susceptibility to cefpodoxime in *Escherichia coli*. *Antimicrobial Agents and Chemotherapy* **46**(12), 3829-836 (2002).

8. Mathlouthi, N. et al. Carbapenemases and extended-spectrum β-lactamases producing Enterobacteriaceae isolated from Tunisian and Libyan hospitals. *The Journal of Infection in Developing Countries* **10**(7), 718-727 (2016).‏

9. Pitout, J. D. D. et al. β-Lactamases responsible for resistance to expanded-spectrum cephalosporins in *Klebsiella pneumoniae, Escherichia coli,* and *Proteus mirabilis* isolates recovered in South Africa. *Antimicrobial agents and chemotherapy* **42**(6), 1350-1354 (1998).‏

10. Ojdana, D. et al. The Occurrence of *bla*CTX-M, *bla*SHV, and *bla*TEM Genes in Extended-Spectrum β-Lactamase-Positive Strains of *Klebsiella pneumoniae*, *Escherichia coli*, and *Proteus mirabilis* in Poland. *Intern J Antibi*, **2014**(1), 935842 (2014).

11. Mirsalehian, A. et al. Detection of VEB‑1, OXA‑10 and PER‑1 genotypes in extended‑spec‑ trum β‑lactamaseproducing *Pseudomonas aeruginosa* strains isolated from burn patients. *Burns* **36**(1), 70–74 (2010).

12. Dallenne, C., Da Costa, A., Decre, D., Favier, C. & Arlet, G. Development of a set of multiplex PCR assays for the detection of genes encoding important B-lactmases in Enterobacteriaceae. *J. Antimicrobe Chemother.* **65**(3), 490-495 (2010).

13. Hou, M. et al. Molecular epidemiology, clinical characteristics and risk factors for bloodstream infection of multidrug-resistant Klebsiella pneumoniae infections in pediatric patients from Tianjin, China. *Infection and Drug Resistance* 7015-7023 (2022).‏

14. Muratani, T., Kobayashi, T. & Matsumoto, T. Emergence and prevalence of β-lactamase-producing *Klebsiella pneumoniae* resistant to cephems in Japan. *International journal of antimicrobial agents* **27**(6), 491-499 (2006).‏

15. Guo, Q. et al. Co-production of SFO-1 and DHA-1 β-lactamases and 16S rRNA methylase ArmA in clinical isolates of Klebsiella pneumoniae. *Journal of antimicrobial chemotherapy* **67**(10), 2361-2366 (2012).‏

16. Sjolander, I. et al. Detection of NDM-2- producing *Acinetbacter baumannii* and VIM – producimg *pseudomonas aeruginosa* in Palestine. *J. Glob. Antimicrobe. Resist.* **2**, 93-97 (2014).

17. Kim, S. Y., Ahn, Y. W., Kim, J. K., Lee, J. H. & Choi, Y. S. Equivalence Analysis for Five Korean NCCP Microorganism Strains as ATCC Reference Strains Alternative in Korean Pharmacopoeia Microbiological Tests. *Yakhak Hoeji* **68**(3), 168-209 (2024).‏

18. Rosato, A. E et al. *mec*A- *bla*Z corepressors in clinical staphylococcus aureus isolates. *Antimicrobial Agents Chemotherapy* **47**, 1463-1466 (2003).

19. Maynard, C. et al. Antimicrobial resistance genes in enterotoxigenic *Escherichia coli* O149: K91 isolates obtained over a 23-year period from pigs. *Antimicrobial agents and chemotherapy* **47**(10), 3214-3221 (2003).‏

20. Randall, L. P., Cooles, S. W., Osborn, M. K., Piddock, L. J. V. & Woodward, M. J. Antibiotic resistance genes, integrons and multiple antibiotic resistance in thirty-five serotypes of *Salmonella enterica* isolated from humans and animals in the UK. *Journal of Antimicrobial Chemotherapy***53**(2), 208-216 (2004).‏

21. Morvan, A. et al. Antimicrobial resistance of *Listeria monocytogenes* strains isolated from humans in France. *Antimicrob. Agents Chemother.* **54**, 2728 –2731 (2010).

22. Odeyemi, A. T., Ayantola, K. J. & Peter, S. Molecular characterization of bacterial isolates and physicochemical assessment of well water samples from hostels at Osekita, Iworoko-Ekiti, Ekiti State. *American Journal of Microbiological Research* **6**(1), 22-32 (2018).‏

23. López, M., Tenorio, C., Del Campo, R., Zarazaga, M. & Torres, C. Characterization of the mechanisms of fluoroquinolone resistance in vancomycin-resistant enterococci of different origins. *Journal of Chemotherapy* **23**(2), 87-91 (2011).‏

24. Cavaco, L. M., Hasman, H., Xia, S. & Aarestrup, F. M. *qnr*D, a novel gene conferring transferable quinolone resistance in *Salmonella enterica* serovar Kentucky and Bovismorbificans strains of human origin. *Antimicrob. Agents Chemother.* **53,** 603–608 (2009).

25. Godreuil, S., Galimand, M., Gerbaud, G., Jacquet, C. & Courvalin, P. Efflux pump Lde is associated with fluoroquinolone resistance in *Listeria monocytogenes*. *Antimicrob. Agents Chemother.* **47,** 704–708 (2003).

26. Toro, C. S. et al. Genetic analysis of antibiotic-resistance determinants in multidrug-resistant *Shigella* strains isolated from Chilean children. *Epidemiology & Infection* **133**(1), 81-86 (2005).‏

27. Mirzaie, S., Faghiri, I., Askari Badouei, M. & Madani, S. A. Molecular detection and occurrence of vancomycin resistance genes (*van* A, B, C1, C2/C3) among *Enterococcus* species isolated from farm ostriches. *Veterinary medicine and science* **9**(1), 226-233 (2023).

28. Van, T. T., Chin, J., Chapman, T., Tran, L. T. & Coloe, P. J. Safety of raw meat and shellfish in Vietnam: an analysis of *Escherichia coli* isolations for antibiotic resistance and virulence genes. *Int. J. Food Microbiol.* **124**(3), 217–23 (2008).

**Supplementary Figure S1. Dendrogram analysis of *Clostridium perfringens* strains (n=289) reveal that *C. perfringens* isolates could be divided into 8 genotypes.**

***
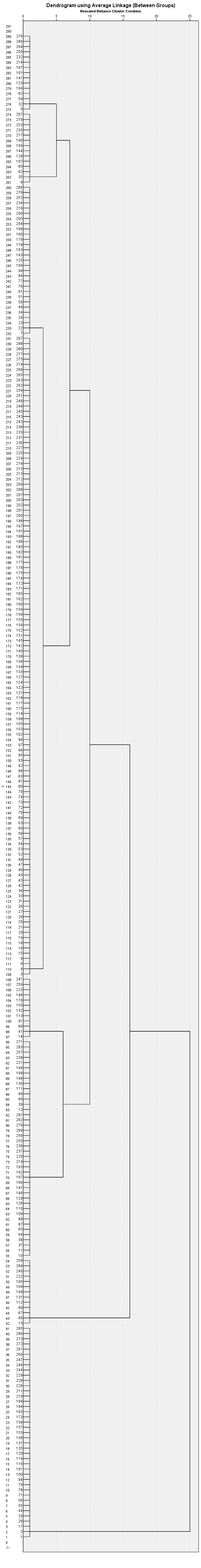
***
